# Supplementary material for: Reproductive Proteins Evolve Faster Than Non-reproductive Proteins Among Solanum Species
Source: Front Plant Sci. 2021 Apr 12;12:635990. doi: 10.3389/fpls.2021.635990 (PMC8072272; doi:10.3389/fpls.2021.635990)

Supplementary Material

Supplementary Text

S1. Ovule tissue RNAseq

Ovule RNAseq was performed on between one to four biological replicates (individual plants) from each of four genotypes (Table S1). Plants were germinated from seed, and cultivated in the IU Biology Greenhouses until flowering.

For each replicate individual, ovules were collected from mature, unpollinated flowers, on the day of anthesis (flower opening). From each flower, individual ovules were separated from ovary tissue using a dissecting needle under a dissecting microscope, and transferred immediately to an Eppendorf collecting tube on dry ice. To obtain sufficient tissue for RNA extraction, ovules were collected from multiple flowers per biological replicate and kept frozen at -80C until extraction. For extraction, all ovule collections per individual were pooled into a single 1.5ml Eppendorf tube, tissue ground using a micropestle under liquid nitrogen, and then total RNA extracted using RNeasy Plant Mini Kits from Qiagen (catalog number 74904), following the manufacturer's protocol. Total RNA was quality checked using an Agilent 2200 TapeStation System prior to library construction. Stranded, paired-end libraries of total RNA were generated from these pools for each sample using Illumina TruSeq Stranded total RNA HT Sample Preparation Kits (Illumina: RS-122-2203), and these libraries were pooled across one lane of Illumina HiSeqTM 2000 (Illumina Inc., San Diego, CA, USA). RNA QC, library preparation and pooling were performed by the Indiana University Center for Genomics and Bioinformatics. Prior to mapping and assembly, reads were trimmed using SHEAR.

S2. Comparing dN/dS using GLMs

Prior to comparing the mean rate of per locus protein evolution (dN/dS) between different classes of loci by fitting generalized linear models (GLMs), we assessed the normality in the distribution of dN/dS using Shapiro-Wilk tests of normality and quantile-quantile plots. In all categories, dN/dS was heavily skewed towards lower values (reflecting the fact that most expressed genes are under purifying selection). To accommodate this in our models, we used a gamma residual distribution with an identity link function, rather than the standard Gaussian assumption. The suitability of assuming a gamma distribution vs a gaussian was inferred from maximum-likelihood estimates of each distribution's parameters against the full dataset of dN/dS estimates using the fitdistrplus R package. The loglikelihood of the maximum likelihood gamma and Gaussian fits was 5626.1 and -3797.6, respectively, indicating that the gamma distribution is a likely a better choice for modeling the distribution of dN/dS across many loci.

S3. Variation in dN and dS among classes of genes, and in comparison to polymorphism within accessions

Because estimates of dN/dS can be influenced by variation in synonymous mutation rates among genes, we also generated and compared separate dS and dN values for our loci. We restricted this comparison to only loci whose sequences were inferred from RNA-seq data (rather than later augmented with available DNA-seq data; Table S3), to ensure the comparison was not influenced by differences in the source (DNA versus RNA) of the data used to generate sequence alignments. We find that, for dS, our estimates are highest for VG loci, intermediate for RP loci, and lowest for GR loci (all contrasts significant; Table S6; Figure S2). For dN, RP loci had the highest estimates, followed by VG and then GR loci; RP loci have significantly higher dN than GR loci (Table S6). We also re-confirm that dN/dS is significantly higher in RP loci compared to GR, and intermediate in VG loci (Table S6). These patterns of variation in dS cannot explain the elevated dN/dS we detect for reproductive loci; in fact, they indicate that we detect this elevated pattern despite slightly elevated synonymous mutation rates in RP loci that should otherwise reduce dN/dS estimates for this class of genes.

In addition, estimates of dN/dS can be influenced by intraspecific polymorphism when this is very high compared to fixed differences between species (see Hahn 2018, Chapter 7). However, estimates of divergence (e.g. dS) across our four species are on the order of 4.0-6.5% (Table S6), which is much larger than estimates of heterozygosity within each of our wild accessions (*S. pimpinellifolium* LA1589: 0.04%; *S. pennellii* LA0716: 0.08%; *S. habrochaites* LA1777: 0.26%; in Table S3 in Pease et al. 2016a). This indicates that most of the SNPs contributing to our estimates of dS, dN, and dN/dS, are fixed differences.

Supplementary Figures

Supplementary Figure 1: Maximum likelihood tree of focal species relationships relative to an outgroup species *Solanum sitiens*. Branch lengths (in average substitutions per site) were obtained from Pease et al. (2016a). Original tree was inferred with RAxML v.8 (Stamatakis, 2014) from a whole-transcriptome concatenated alignment, using the GTRGAMMA approximation of the general time reversible model of substitution, allowing rate heterogeneity.

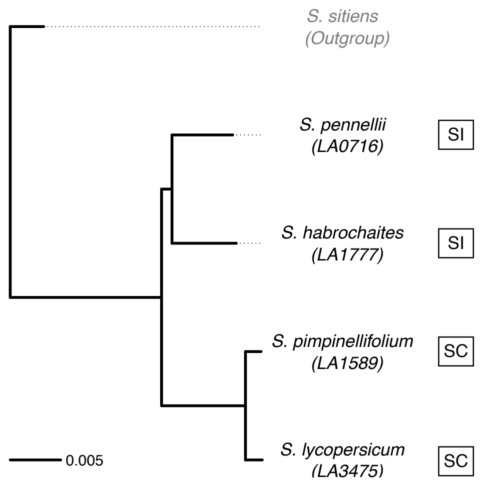

Supplementary Figure 2: Distributions of inferred per-locus dN (left), dS (middle), and dN/dS (omega) (right) from each broad class of loci: RP (upper panels), GR (middle panels), and VG (lower panels). Supporting analyses are in Table S6.

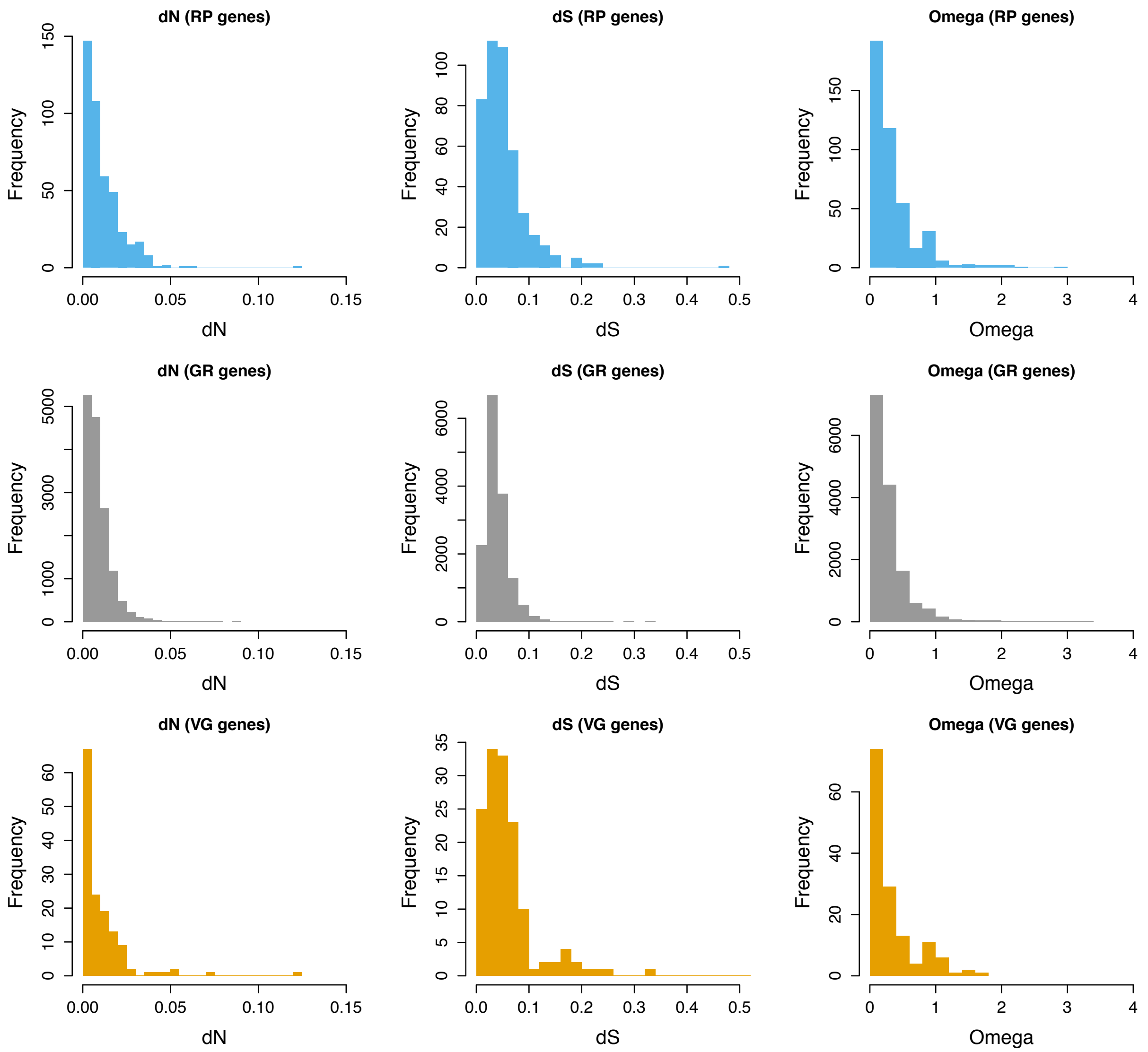

Supplementary Figure 3: Relationship between per locus mean level of gene expression (TPM) and per locus protein evolution (dN/dS) in loci expressed in (left) general/GR, (middle) reproductive/RP, and (right) vegetative/VG tissues in Solanum species. Each point indicates an individual locus. Regression lines shown in black; 95% CI shown in grey. (Note that the confidence intervals around the regression line for GR loci are narrow.) Supporting analyses are in Table S13.

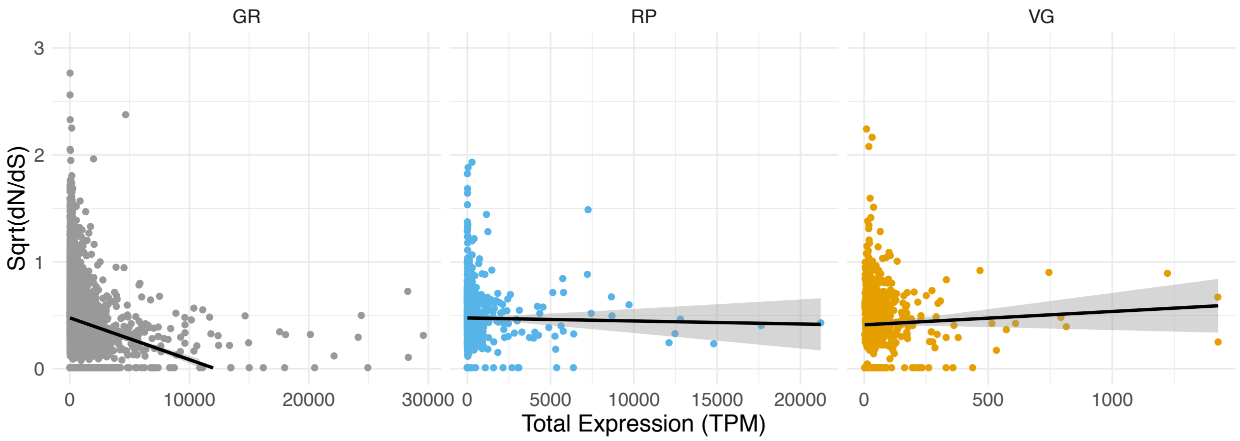

Supplement: Supplementary file 1 [file Data_Sheet_1.PDF]
